# DAG-Based Blockchain Sharding for Secure Federated Learning with Non-IID Data

**DOI:** 10.3390/s22218263

**Published:** 2022-10-28

**Authors:** Jungjae Lee, Wooseong Kim

**Affiliations:** Computer Engineering Department, Gachon University, Seongnam 1342, Korea

**Keywords:** blockchain, federated learning, smart contract, model-poisoning attack

## Abstract

Federated learning is a type of privacy-preserving, collaborative machine learning. Instead of sharing raw data, the federated learning process cooperatively exchanges the model parameters and aggregates them in a decentralized manner through multiple users. In this study, we designed and implemented a hierarchical blockchain system using a public blockchain for a federated learning process without a trusted curator. This prevents model-poisoning attacks and provides secure updates of a global model. We conducted a comprehensive empirical study to characterize the performance of federated learning in our testbed and identify potential performance bottlenecks, thereby gaining a better understanding of the system.

## 1. Introduction

Machine learning has evolved over the last few decades, and deep learning based on multilayered deep neural networks (DNNs) has received an enormous amount of interest in various areas of research [1,2,3]. In particular, deep learning using convolutional neural networks (CNNs) has demonstrated a notable performance in terms of object detection and classification used in computer vision.

Despite the remarkable performance achieved by deep learning, concerns related to data privacy have increased, particularly in the medical field, which requires private and sensitive patient information for machine learning. For this purpose, general data protection regulations (EU GDPR) [4] were recently released.

To achieve privacy protection, Google proposed federated learning, which enabled collaborative learning without the sharing of private data samples [5,6]. Instead, learners exchange weights or gradient parameters of their own local model for updating. In practical terms, a trusted central worker as a curator aggregates models of multiple learners and returns an aggregated model as a joint representative model.

For the curator, blockchain is a popular consideration as a method to securely protect the joint model from poisoning attacks [7]. Federated learning is vulnerable to model-poisoning attacks [8,9,10], which are more significant than data poisoning. Malicious nodes can easily distort the model parameters and degrade the agent’s intelligence. Therefore, several works have been proposed for robust aggregation. Blanchard et al. [11] proposed the Krum algorithm, which updates the global model by a majority group with a minimum sum of Euclidean distances among local models of members. Similarly, Yin et al. [12] proposed the median and trimmed mean algorithms, which remove extreme local gradient values to ensure the robustness of the distributed gradient descent algorithms. Recently, a clustering approach to remove malicious local gradients has been introduced [13,14]. Li et al. [15] introduced a central server framework to detect and remove malicious model updates using a powerful detection model. However, aforementioned approaches that rely on the central sever for the aggregation are vulnerable to additional security threats. Che et al. [16] proposed a committee framework for the Byzantine robust update that guarantees convergence.

Recent successes in cryptocurrencies, such as Bitcoin [17], Ethereum [18], Ripple [19], and Stellar [20], have proven that the blockchain consensus algorithms (e.g., proof of work) can prevent Sybil attacks with invalid and malicious transactions. Therefore, the blockchain can be used for a serverless FL framework. Based on its consensus algorithm, the model parameters have been updated and distributed to users after a cross-verification [21,22].

Several previous studies have explored a collaboration between blockchain and federated learning. The block rate and interval of the blockchain are critical for updating the model through federated learning. Kim et al. [23] introduced a seminal study on blockchain-based federated learning (BlockFL) in terms of the architecture and analysis of the end-to-end latency. Pokhrel et al. [21] conducted a delay analysis of BlockFL in wireless and mobile vehicular networks using a mathematical model. Lu et al. [24] proposed asynchronous federated learning for selecting a subset of heterogeneous vehicles of a global model considering their computing power and communication delay. They reduced the learning delay for the combinatorial optimization problem using a deep reinforcement learning (DRL) algorithm. Additionally, they improved the communication efficiency and latency of the blockchain for federated learning in mobile edge networks [25,26]. Desai et al. [27] implemented and experimented with blockchain-based federated learning on the hyperledger fabric. Moreover, model-poisoning attacks have been explored in several studies. Weng et al. [28] proposed an incentive mechanism using the blockchain for federated learning, which prevents misbehavior of the participants. Additionally, Qu et al. [29] analyzed a blockchain that can protect a global model from a poisoning attack, which results in the blockchain growing sufficiently fast in terms of the poisoning resistance.

However, reaching a consensus for an aggregated model is a challenge if federated learning has non-independent and identically distributed (non-IID) datasets across different users. This causes a skewness of the model parameters, eventually decreases the accuracy, and disturbs the convergence. Several studies have been conducted to solve the non-IID data problem in federated learning, considering solutions such as data sharing, node clustering, and algorithmic approaches [30]. Some studies have considered the use of the blockchain to address this problem. Using the blockchain and DRL for finding top-K clustered clients for the blockchain update, Wang et al. [31] solved the divergence problem that occurs from the non-IID characteristic of distributed datasets. Li et al. [32] proposed committee-based cross-verification for global model updates, which is effective against non-IID datasets and poisoning attacks.

Although several studies have discussed the challenge of non-IID datasets and model poisoning, the proposed solutions have limitations in that users have feasible test data for verifying and obtaining a consensus regarding the global model. However, users with parts of a dataset used for training have a high possibility of having the same data for testing.

To solve the non-IID and model-poisoning problems, we propose two-level blockchains for local model aggregation with distributed verification in directed acyclic graph (DAG)-based blockchain shards and global model aggregation by shard committees in the main blockchain. The major contributions of this study are highlighted below:

- This is the first blockchain study on model protection from a poisoning attack during federated learning with non-IID train and test datasets.
- A novel secure update mechanism of the global model is proposed from the asynchronously operating distributed DAG shards.
- This is an empirical study conducted on a real testbed for investigating the feasibility of the proposed system using a convolutional neural network (CNN) and the MNIST dataset.

In our testbed, we evaluated the local model update driven by our novel tip selection algorithm in the DAG shards as well as global model aggregation based on the proposed voting system. The tip selection algorithm leads the model to select a tip based on the similarity and multiplicity of the tip’s model in the tangle of model transactions. Experimental results show robust learning performance even with 50% malicious nodes, such as 85.24%, 81.53%, and 77.16% detection accuracy for model-poisoning, data-poisoning and label-swapping attacks, respectively.

The remainder of this paper is organized as follows. We briefly review federated learning and blockchain combinations in Section 2 and Section 3, respectively. We then describe our proposed system in Section 4 and present the experimental results in Section 5. Finally, we present some concluding remarks regarding this study in Section 6.

## 2. Federated Learning

### 2.1. Federated Model Averaging

Federated learning enables users to update a joint global model using their own private data iteratively. For this purpose, a curator selects a subset of connected workers and provides them with the latest global model for local training. Subsequently, the curator collects and aggregates the local models updated by the workers for a new global model.

Federated learning can be formulated as the following joint optimization problem. Each node, i∈N, owns a set of local data samples, $D_{i}$, and trains a model to solve a regression problem using such samples in a distributed manner. Using a sample d=(x,y), the training objective is to minimize a loss function such as the mean square error (MSE), where $l\left(\omega ;d\right)={\left(x_{d}^{⊤}\omega -y_{d}\right)}^{2}$. For all nodes N, the joint optimization problem can be written as follows:

(1) $$\min_{\omega }f\left(\omega \right)≜\frac{1}{\left|D\right|}\sum_{i\in N}D_{i}F_{i}\left(\omega \right),$$

where the loss function $F_{i}\left(\omega \right)≜\frac{1}{D_{i}}\sum_{d\in D_{i}}l\left(\omega ;d\right)$ and $D=\cup_{i=1}^{N}D_{i}$.

To solve the above distributed optimization problem, the global model can be updated as $\omega^{\left(t+1\right)}\leftarrow \omega^{\left(t\right)}-\eta \nabla f\left(\omega^{\left(t\right)}\right)$ using gradients of nodes based on a stochastic gradient descent (SGD) as follows:

(2) $$\nabla f\left(\omega^{\left(t\right)}\right)=\sum_{i\in N}\frac{D_{i}}{\left|D\right|}\nabla F_{i}\left(\omega^{\left(t\right)}\right)$$

Instead of training with every sample and communicating with its gradient, each node can update the local model based on the mini-batch and send its own $\omega_{i}^{\left(t+1\right)}$ to the curator infrequently. For this, the global model is updated simply by federated averaging (FedAvg) at each epoch, *t* of $D_{i}$ samples, as follow [33]:

(3) $$\omega^{\left(t+1\right)}\leftarrow \sum_{i\in N}\frac{D_{i}}{\left|D\right|}\omega_{i}^{\left(t\right)}$$

### 2.2. Asynchronous Federated Learning

Because the local training delay varies according to the capability of the worker devices, the central curator needs to select a set of workers for aggregation to avoid excessive update delays from the stragglers [24]. Alternatively, several studies [34,35,36] have explored asynchronous federated learning instead of conventional synchronous learning.

Cong et al. [34] proposed *FedAsynch* to average new weight parameters, ω, adaptively by a mixing parameter α∈(0,1), which is determined through the decreasing staleness function $f_{s}$ and constant variable τ, $\omega^{\left(t+1\right)}=\left(1-\alpha \right)\omega^{\left(t\right)}+\alpha \omega_{i}^{\left(t\right)}$, where $\alpha =f_{s}\left(t-\tau \right)$. Accordingly, the old model parameters are slightly added using the smaller α. Chen et al. [36] investigated online aggregation, where the curator and workers exchange and update the models asynchronously. Similarly, they adopted a decaying local update with a β coefficient that induces a balance between the old and new local gradients. Chai et al. [35] proposed *FedAT*, which aggregates a global model with a local model of a cluster composed of homogeneous devices.

These previous studies proved mathematically that asynchronous updates can achieve a learning convergence with a decaying coefficient for the weight average of the models. Additionally, the experimental results showed that a feasible level of accuracy can be achieved even with non-IID data for local training.

### 2.3. Training with Non-IID Data

Federated learning based on a stochastic gradient follows IID sampling to avoid biased estimates of the gradient for the global model. However, it is unrealistic to obtain local IID samples for all distributed worker devices. Although *FedAvg* is known to operate even with non-IID data, it degrades the accuracy by 11% for MNIST compared to an IID case [37]. The degree of weight divergence owing to the use of non-IID data is significant based on the data skewness of a node in comparison to the actual distribution for the whole population.

The convergence of *FedAvg* on non-IID data was analyzed in [38]. The required aggregation rounds for a particular accuracy are the number of steps required for the loss *e*, i.e., $T_{e}$ over *E* local iterations,

(4) $$\frac{T_{e}}{E}\propto \left(1+\frac{1}{\left|N\right|}\right)EG^{2}$$

where the required number of aggregation rounds is proportional to the inverse of |N|, *E*, and the upper bound of the gradient norm $G^{2}$, i.e., $O(\frac{EG^{2}}{\left|N\right|})$. In other words, a large number of nodes and limited local iterations contribute to improving the accuracy of a global model, whereas heavy local iterations, such as $10^{3}$, cause a weight divergence and decrease the accuracy. Even with IID data, the weight divergence is likely significant if local models have different initial parameters [37].

In a survey conducted on *FedAvg* with non-IID data [30], data sharing, fine-tuning algorithms with personalized models, and client clustering approaches were discussed. Here, we focused on a clustering approach that creates node groups for federated learning according to their distribution of attributes. These attributes can be mutually exclusive across clients or partially overlap.

## 3. Blockchain for Federated Learning

### 3.1. Blockchain Overview

Blockchain is a tamper-proof ledger operating in decentralized and anonymous peer-to-peer (P2P) networks. Individuals and organizations use the blockchain to record their transactions without requiring permission to access it. For this purpose, the transactions are verified across participants before they are written in chained blocks. The Nakamoto consensus algorithm, i.e., proof of work (PoW) for verification, is the most famous algorithm for Bitcoin, and although it works effectively for protection from Sybil attacks and data manipulation, it is expensive because of the required hash power and long block interval.

Many other PoX algorithms have emerged, including proof of stake (PoS) (e.g., Peercoin [39], Blackcoin [40], and follow-the-Satoshi procedure [41,42]). To accelerate this agreement, a delegated proof of stake (DPoS) was proposed [43]. Additionally, proof of burn [44], proof of validation [45], proof of elapsed time, and proof of capacity (e.g., storage space) [46,47] have been proposed.

### 3.2. Blockchain Scalability

Distributed blockchain systems suffer from intrinsic scalability problems because they synchronize a single chain globally without forking [48,49,50,51]. For instance, Bitcoin can process only seven transactions per second (TPS) compared to legacy systems such as VISA, which can tolerate 2000 TPS. There have been several approaches to scaling up the blockchain.

First, off-chain techniques such as a Raiden network [52] and plasma [53] are used to offload transactions in the main chain. Additionally, L2 protocols are needed to verify the state information between the local and global main chains periodically or aperiodically, which causes an update latency between them [54]. Second, multiple on-chains, called blockchain shards, process transactions in parallel using multiple committees [48]. Several sharding-based blockchains have been proposed, including RScoin [55], omniLedger [56], and RapidChain [50].

Instead of a legacy chain structure, a directed acyclic graph (DAG) has recently been considered to overcome the scalability problem [57]. Some cryptocurrencies based on the DAG blockchain have already been developed, including NXT [41], IOTA [58], and dagCoin [59]. The DAG structure is advantageous for processing multiple transactions independently, where participants select tips to add their own transactions and verify previous transactions along random walk paths. Such a tip selection algorithm enables nodes to find a reliable path that has been evaluated much earlier by following the transactions. This tangle can create multiple reliable chains within the DAG, as indicated by a higher cumulative weight. For DAG growth, the tip selection range should be configured that prevents the DAG structure from becoming wider as the same tips are selected heavily.

In this study, we consider a DAG-based blockchain for federated learning that allows nodes to share local models securely and update them asynchronously in a distributed environment.

### 3.3. Blockchain for Federated Learning

Seminal studies on the combination of blockchain and federated learning have mostly investigated the effects of block creation and consensus delay on federated learning procedures [21,23,25,26]. Subsequently, studies on asynchronous and distributed updates in federated learning have been based on blockchain [24,60].

In federated learning, it is critical to prevent model poisoning by anonymous adversarial nodes. Consensus algorithms of the blockchain, such as PoW [29] and PoS [61], can efficiently protect the global model from poisoning attacks by malicious nodes. Several studies [25,62] have considered private and permissioned blockchains, in which trusted nodes manage the learning procedure. Otherwise, reliable nodes can be recruited for the consensus procedure based on their reputations [63] or incentive systems [28]. For example, in [32], reliable committee members are elected for cross-verification of the global model using incentives for honest behavior. However, the private blockchain limits its usability, and alternative approaches require additional systems, such as cryptocurrency and reputation scores.

## 4. DAG-Based Sharding for Secure Federated Learning

In this section, we present DAG-based sharding for federated learning under poison attacks, such as model poisoning, data poisoning, and label flipping. In a distributed learning environment, the expected challenges are enumerated as follows:

- Each node has a non-IID dataset for training and testing with a different distribution probability.
- The computing capability of a node varies according to the device type.
- Open accessibility allows Sybil-based poisoning attacks on local or global models.

Previous studies on algorithmic or systematic approaches used to solve non-IID datasets and asynchronous update problems assume that each device has IID test sets for conducting an evaluation on an aggregated model. However, it is unrealistic for nodes to have a sufficient number of samples to estimate the loss of the global model. Therefore, a consensus based on the majority is a possible way to update the global model [32].

Unfortunately, a simple voting solution cannot prevent model poisoning with targeted attacks because nodes cannot recognize the target attack if the targeted part of the model is irrelevant to their own data set. For instance, nodes who only have image data 2 and 3 of MNIST will vote for a poisoned model with 90% accuracy for data 2 and 3 rather than the normal model with 85% accuracy. However, the chosen model probably has lower accuracy for other image data, which can happen even in untargeted attacks on the model.

Therefore, we propose a two-step verification procedure for the validation of aggregated models using hierarchical blockchains that consist of the main public blockchains, such as Bitcoin and Ethereum, and local shards based on DAGs. Details of the proposed system are described in the following subsections. Related symbols are described in Table 1.

### 4.1. Hierarchical Blockchain Architecture for Federated Learning

**Figure 1:**
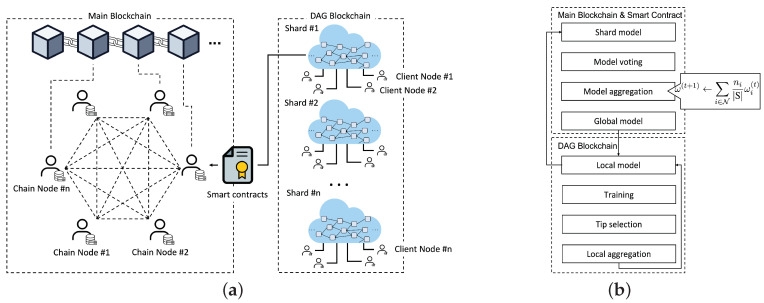
System architecture and flow. (**a**) System architecture for federated learning with hierarchical blockchains; (**b**) System flow of hierarchical blockchains.

The hierarchical blockchains for secure federated learning are illustrated in Figure 1a, where multiple DAG-based local shards are autonomously established by the participants for local model aggregations. Additionally, a public blockchain is used for global model aggregation by all users.

As illustrated in Figure 1b, model verifications and updates are iteratively achieved through a closed loop between the main blockchain and its shards. In a local shard chain, the local model is updated asynchronously according to the computing capability of each node. The local model of the shard is periodically uploaded for global aggregation on the main chain. For example, nodes of shard #1 create model transactions and combine them locally every *n* epochs in the DAG and upload a final local model to the main blockchain after reaching *m* epochs. Subsequently, one of the chain nodes in the main blockchain sequentially aggregates the local models. Although the number of epochs for the local model update can vary, the total number of local iterations *E* in Equation (4) for global aggregation is comparable for all shards.

### 4.2. DAG-Based Shard Formation

For local training with non-IID data, data-oriented blockchain shards are established autonomously by nodes according to their data distribution. For instance, node *i* joins shard *s* with the smallest cross-entropy $-\sum_{x}p_{i}\left(x\right)\log_{2}p_{s}\left(x\right)$, where $p_{i}\left(x\right)$ and $p_{s}\left(x\right)$ are the probability distributions of node *i* and shard *s* for data *x*. However, owing to privacy concerns, the sample distribution is typically unknown to other nodes.

Instead, each worker selects a shard based on the local model accuracy evaluated by its own data, i.e., a shard with the highest accuracy model. As the training and aggregation are repeated, the best shard for a node can be changed. Because a local shard is a permissionless DAG chain, the nodes can join and leave the shards liberally according to their own accuracy.

Initially, each node trains a local model during *n* epochs and broadcasts it to the nearby nodes. Once the nodes receive other node models by flooding, they form |S| shards using the following the K-means clustering algorithm, which determines sets $S_{k}$ iteratively to minimize the sum of the weight difference.

(5) $$\underset{S}{arg min}\sum_{k=1}^{\left|S\right|}\;\frac{1}{|S_{k}|}\;\sum_{\omega_{i},\omega_{j}\in S_{k}}{∥\omega_{i}-\omega_{j}∥}^{2}$$

The genesis model of shard #*i* is the closest to the centroid from K-means clustering as follows:

(6) $$\underset{\omega_{i}}{arg min}{∥\omega_{i}-\frac{1}{\left|S_{k}\right|}\sum_{\omega_{j}\in S_{k}}\omega_{j}∥}^{2}$$

The Algorithm 1 describes the shard formation process in detail. In lines 1–11, each node creates a model using its own dataset. Lines 12–17 indicate the initial process for client nodes to create a shard. First, each client node exchanges models and their corresponding node information with each other. Each node then creates shards cooperatively based on the clustering algorithm in Equations (5) and (6). In lines 19 and 20, the client node continuously switches shards based on the accuracy during the learning process.
**Algorithm 1** Shard initiation algorithm.**Input:**|N|≥|S|1:**procedure**engage shard(r,|S|)2:    **if** *r* = 0 **then**3:        Initialize $w_{0}$4:        **for** each client node i∈N **do**5:           B← Split $D_{i}$ by batches of size *B*6:           **for** local epoch e=1,2,⋯,E **do**7:               **for** batch b∈B **do**8:                   $\omega_{i}^{\left(t+1\right)}\leftarrow \omega_{i}^{\left(t\right)}-\eta \nabla f\left(\omega_{i}^{\left(t\right)}\right)$9:               **end for**10:           **end for**11:        **end for**12:        **while** each client node *i* participate in shard $S_{k}$ **do**13:           Broadcast $\omega_{i}$ into federated learning network14:           Receive ω from other nodes15:           $S_{k}\leftarrow$ K-means clustering(ω,|S|)16:           node *i* participate in a shard $S_{k}$17:        **end while**18:    **else**19:        Select highest accuracy $M_{m}^{t}$20:        node *i* switches into a shard $S_{m}$21:    **end if**22:**end procedure**

### 4.3. Local Model Aggregation

For the local model aggregation in a DAG-based shard, the tip selection algorithm adopts a two-phase procedure: (i) selecting candidate tips at least more than |M|=2 based on model accuracy and similarity, and (ii) performing a check of the aggregation history for multiplicity using a random-walk exploration.

(7) $$\min_{m}\;\frac{1-\alpha }{|M|+1}F_{i}\left(\tilde{\omega }\right)+\alpha \sum_{m\in M}\left(1-\frac{\omega_{m}\cdot \omega_{i}}{\|\omega_{m}{\|}_{2}{\left\|\omega_{i}\right\|}_{2}}\right)$$

where $\tilde{\omega }=\sum_{m\in M}\omega_{m}+\omega_{i}$ and $F_{i}\left(\tilde{\omega }\right)$ is the loss estimated by the test samples, as in Equation (1), in which *m* is a transaction that contains a local model.

The right-hand term of this equation is the cosine similarity of the NN weight parameters. Additionally, α∈(0,1) is the weight value for multiple objectives. Each node selects tips with a higher accuracy for its own test data and similarity. Owing to the limitations of an accuracy evaluation with the skewed test samples, the model similarity information is useful for preventing a model divergence and targeted attacks on the model parameters.

Since each node commits a single transaction during the average epoch time $E[T_{ep}]$, the search space for the tips is limited by the number of nodes in a shard. Otherwise, a deep search space imposes a higher overhead on evaluating the level of accuracy and similarity. To approximate the number of shared nodes, a node can estimate the mean of the arrival rate of the transactions, λ, and configure the search space as $\lambda E[T_{ep}]$.

**Figure 2:**
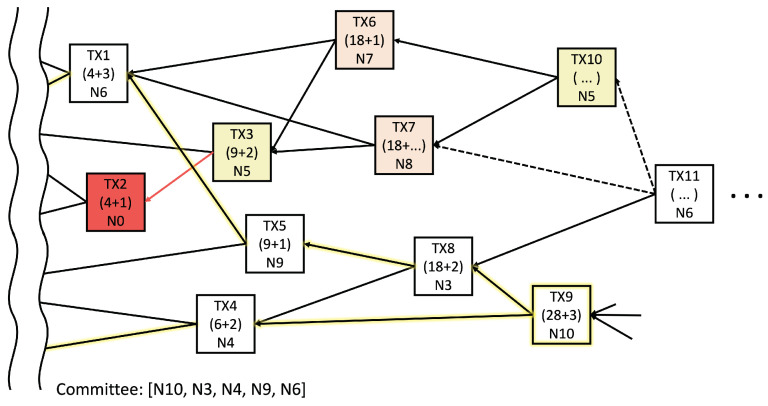
Example of tip selection: the white transaction, a normal model; the red transaction, a poisoned model; the other color transaction, a model contaminated by aggregating a poisoned transaction.

Owing to the heterogeneity of the computing power, a different number of transactions from each device may occur during the same epochs. This causes the local aggregation to be biased toward a model of powerful devices. Additionally, malicious users can generate meaningless models to destroy the local model. Accordingly, to check the aggregation history, our tip selection algorithm walks randomly based on the Markov chain Monte Carlo selection algorithm (MCMC) [64], and the transition probability from transactions *j* and *k* can be derived through a monotonically increasing function.

(8) $$P_{j,k}=\log\left(\left(\zeta_{j}-\zeta_{k}\right)-\sigma \right)$$

where σ is a positive constant for normalization, and $\zeta_{j,k}$ is the cumulative reference score (CRS) of a transaction. The CRS is the cumulative value of the reference score (RS), which indicates how many transactions refer to aggregation. Figure 2 illustrates an example of a transaction tangle in a DAG shard, where the first number in each transaction is the CRS and the second number is the RS. Additionally, N# denotes the identifier of the transaction node. Random walking from a candidate tip allows a node to find a reliable aggregation path for the local model (highlighted by yellow edges).

Supposing that a poisoned model colored in red appears in TX2, some of the nodes will likely choose TX2 as a tip owing to a lack of information, i.e., TX3 is colored yellow. Subsequently, the poisoned model can be propagated to the following models even though the degree of distortion can be diminished. For example, the colored TX6 and TX7 mix the contaminated TX3 with another fine model in the figure. Unfortunately, because the following transactions prefer to select the contaminated model owing to the similarity, such distortion from the wrong model combination continues. Supposing that TX3 and TX10 are issued by the same node, TX10 is likely to select TX6 and TX7 over the others because of their high cosine similarity. Therefore, the multiplicity of the transactions as a degree of model duplication must be considered along the random walk path. A higher multiplicity path eventually reduces the model diversity and final accuracy.

The Algorithm 2 describes the local model aggregation algorithm in detail. Lines 2–6 configure the genesis model with the global model generated in the previous round. Recall that a node selects tips for local aggregation in two steps: lines 13–19 represent the first step in calculating the transaction score within the search space $\lambda E[T_{ep}]$ based on Equation (7). In lines 20 and 21 as the second phase, the two transactions with the lowest multiplicity values are selected. In this study, we considered two tips for local aggregation. MULTI() is a function that derives the number of transactions of the same node along a random path using the MCMC selection algorithm for a given tip.
**Algorithm 2** Local model aggregation algorithm.**Input:** node i∈N has participated in $S_{k}$
1:**procedure**local aggregation(*r*)2:    **if** *r* = 0 **then**3:        Initialize $w_{0}$4:    **else**5:        w← weight of global model from previous round6:    **end if**7:    B← Split $D_{i}$ by batches of size *B*8:    **for** local epoch e=1,2,⋯,E **do**9:        **for** batch b∈B **do**10:           $\omega_{i}^{\left(t+1\right)}\leftarrow \omega_{i}^{\left(t\right)}-\eta \nabla f\left(\omega_{i}^{\left(t\right)}\right)$11:        **end for**12:    **end for**13:    **for** *m*∈$M(\lambda E\left[T_{ep}\right])$ **do**14:        $R_{m}$$\leftarrow \min_{m}\;\frac{1-\alpha }{|M|+1}F_{i}\left(\tilde{\omega }\right)+\alpha \sum_{m\in M}\left(1-\frac{\omega_{m}\cdot \omega_{i}}{\|\omega_{m}{\|}_{2}{\left\|\omega_{i}\right\|}_{2}}\right)$15:        $C_{m}$ ← *∞*16:        **if** $R_{m}$ > E[R] **then**17:           $C_{m}$← MULTI(*m*)18:        **end if**19:        $\omega_{n},\omega_{m}\leftarrow$ min $C_{m},C_{n}$, m,n∈M20:    **end for**21:    $\omega^{\left(t+1\right)}\leftarrow \sum_{i\in W}\frac{D_{i}}{\left|D\right|}\omega_{i}^{\left(t\right)}$ where *W* is $\left\{\omega_{m},\omega_{n},\omega_{i}\right\}$22:**end procedure**

For local updates, a node trains its own model, adds it to one of the DAG shards, and broadcasts the DAG chain over the network. In contrast to a conventional chain-based shard that suffers from a serialization delay owing to concurrent transactions, a DAG-based shard allows heterogeneous devices to record their own models asynchronously and update independently. This asynchronous local update leads to the avoidance of aggregation delay from stragglers.

### 4.4. Global Model Aggregation

At the end of a round, the local model with the highest RS is recommended as a final model for global aggregation since the best local model probably receives more selections from others. Here, the corresponding transaction should have CRS around $\left[\left(1-\frac{1}{\left|M\right|}\right){\mathrm{CRS}}_{max},{\mathrm{CRS}}_{max}\right]$ to guarantee enough local aggregation within the shard. To report the final model to the main blockchain, the transaction owner as a leader of the shard creates a new transaction for their own model and broadcasts it to the main blockchain through the chain nodes for global aggregation, as illustrated in Figure 1a.

A special chain node, which plays the role of global model management, aggregates local models recorded in the main blockchain; otherwise, each node aggregates the models of all shards individually. Furthermore, the global model can be recorded using a special chain node in the main blockchain for other users.

However, it is critical that malicious nodes upload a poisoned local model in the main blockchain because the chain node cannot validate the uploaded local models. For this, we adopted a voting system based on the DAG shard in which the leader of each shard confirms the voting members who contribute to the final model. In practical terms, the leader attaches the DAG path information (i.e., the sequence of local transaction numbers) of the final model to the global transaction. The leader registers the voting members using a smart contract with their public keys, which can authorize voting from the members using their signatures.

**Figure 3:**
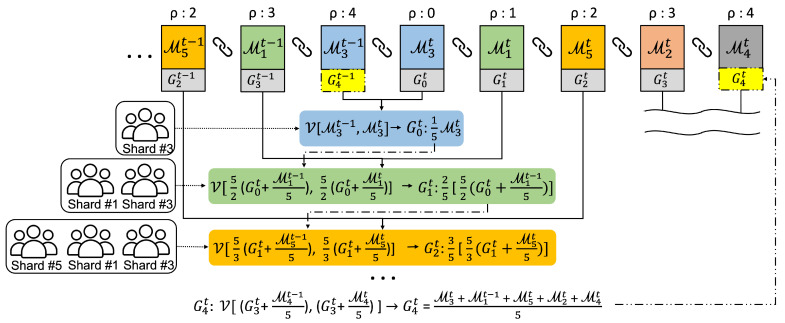
Example of global model aggregation.

Figure 3 describes the aggregation procedure in the main blockchain when five shards(#1 ∼ #5) exist in the federated learning network, where the shard local models are uploaded asynchronously during the round. Thereafter, the global model $G_{i}^{t}$ for round *t* is updated sequentially whenever the local models arrive. The voting members evaluate the global model using their own dataset to find the most accurate model across all datasets. Additionally, we compare the old and new local models before global aggregation because the selected local model might be contaminated after the last round, as illustrated in Figure 2.

In the example of the figure, local models are uploaded in order of shards #3, #1, #5, #2, and #4, and ρ indicates the sequence index. First, the committee members of a shard *s* vote between the new $M_{s}^{t}$ and old $M_{s}^{t-1}$ that were uploaded in the previous round once the local model $M_{s}^{t}$ appears on the main blockchain. For instance, when the local model $M_{3}^{t}$ is uploaded from the shard #3, the committee members vote between $M_{3}^{t}$ and $M_{3}^{t-1}$. Accordingly, the first new global model $G_{0}^{t}$ is $M_{3}^{t}$ if $M_{3}^{t}$ wins during the voting process. Next, $M_{1}^{t}$ comes from the shard #1, and then voting is conducted again between $G_{0}^{t}+M_{1}^{t-1}$ and $G_{0}^{t}+M_{1}^{t}$, where the weight of each model is multiplied by 5/2 in order to match the weight ratio for the two models. Additionally, the voting members are all of shard committees #1 and #3. If the voting result is $G_{0}^{t}+M_{1}^{t-1}$, $G_{1}^{t}$ becomes $G_{0}^{t}+M_{1}^{t-1}$. Similarly, the following aggregations for the local models of the shards #5, #2, and #4 are conducted. Finally, this aggregation procedure yields a new global model $G_{4}^{t}$ at ρ=4 from the old $G_{4}^{t-1}$, which will be a basis model for training in the next round.

The generalized equation for the above global aggregation is as follows:

(9) $$G_{\rho }=\frac{\rho +1}{\left|S\right|}\left[V\left(\frac{\left|S\right|}{\rho +1}\left(G_{\rho -1}+\frac{M_{s}^{t-1}}{\left|S\right|}\right),\;\frac{\left|S\right|}{\rho +1}\left(G_{\rho -1}+\frac{M_{s}^{t}}{\left|S\right|}\right)\right)\right],$$

where V indicates a voting function, i.e., nodes belonging to the committee select one model based on the accuracy of the two models. ρ is an integer value starting from 0 to |S| in order of the shard models uploaded to the main blockchain. Accordingly, a total $G_{|S|-1}$ global model will be created.

Algorithm 3 describes the global model aggregation procedure. The FORM COMMITTEE function for generating a committee starts from the final local model and searches for transactions through the tip selection algorithm. Figure 2 shows an example in which the committee is generated using the FORM COMMITTEE function. If TX9 is selected as the final model, the highlighted edges indicate a reliable aggregation path for the final model. Thus, the committee members can be (N10, N3, N4, N9, N6). These committee members vote for the global models through the procedure in lines 22–29, until the local models are uploaded from all shards. After the new global model is created, it is updated through line 30 to ensure the training stability; additionally, β∈(0,1) is a ratio parameter between the old and new global models.
**Algorithm 3** Global model aggregation algorithm.1:**procedure**form committee(start TX)2:    nodes = []3:    **while** true **do**4:        **if** next TX = genesis TX **then**5:           return nodes6:        **else if** start TX **then**7:           select two TX8:        **else**9:           Select the next TX that has higher CRS between two TXs.10:        **end if**11:        nodes← node(TX)12:    **end while**13:**end procedure**14:**procedure**generate global model15:    ρ=016:    **for** s∈S **do**17:        $M_{s}^{t}\leftarrow \mathrm{TX}\;\mathrm{with}\;\mathrm{the}\;\mathrm{highest}\;\mathrm{RS}\;\mathrm{among}\;\mathrm{the}\;\left[\left(1-\frac{1}{\left|M\right|}\right){\mathrm{CRS}}_{max},{\mathrm{CRS}}_{max}\right]\mathrm{in}\;s$18:        $C_{s}\leftarrow$ FORM COMMITTEE($M_{s}^{t}$)19:        $M_{s}^{t-1}\leftarrow \mathrm{Get}\;\mathrm{model}\;\mathrm{from}\;\mathrm{main}\;\mathrm{blockchain}$20:        **if** ρ=0 **then**21:           $G_{0}^{t}=V\left[M_{s}^{t-1},M_{s}^{t}\right]$ by $\cup_{k}^{S}C_{k}$22:        **else**23:           result = $V\left(\frac{\left|S\right|}{\rho +1}\left(G_{\rho -1}+\frac{M_{s}^{t-1}}{\left|S\right|}\right),\;\frac{\left|S\right|}{\rho +1}\left(G_{\rho -1}+\frac{M_{s}^{t}}{\left|S\right|}\right)\right)$ by $\cup_{k}^{S}C_{k}$24:           $G_{\rho }^{t}=\frac{\rho +1}{\left|S\right|}\;\mathrm{result}$25:        **end if**26:        ρ+=127:    **end for**28:    $G^{\left(t+1\right)}=\left(1-\beta \right)G^{\left(t-1\right)}+\beta G^{\left(t\right)}$29:**end procedure**

## 5. Experiment

### 5.1. Experimental Setup

We implemented our proposed system using the Ethereum blockchain and conducted an experiment with 50 client nodes for federated learning. Five DAG-based shards were realized, and a special chain node was developed to execute a smart contract for the voting procedure. Solidity script codes were used for the smart contract.

The federated learning model was a LeNet5 [65] model of a convolutional neural network (CNN) having three convolutional layers, two max pooling layers, and one fully connected layer with an output layer. We used the Adam optimizer and the cross-entropy loss function. The learning rate was set at 0.0001. The batch size of each epoch was 64, and each round had five epochs. Owing to the heterogeneity of the devices, each node invoked an average of four model transactions during a round.

For the experiment, the MNIST dataset was used, which is an image dataset of handwritten numbers from 0 to 9. It consists of 60,000 training and 10,000 test datasets. For the federated learning study with a non-IID dataset, the dataset was configured as mutually exclusive across the clients. Each node had only two types of data among a possible 10 and randomly selected 10 ∼ 90% of the image data of each class. For instance, a node had no data for other classes 2 to 9 if it chose 0 and 1. The test dataset was configured in the same manner as the training dataset. In other words, a node on each shard had two separate data classes; therefore, all data classes of the MNIST were covered across the five shards.

#### Attack Scenarios

The following three attack scenarios were considered for performance evaluation:

- Model-poisoning attack: The purpose of a model-poisoning attack is to upload an arbitrarily altered model to deviate the global model from a target model. In our experiments, a malicious node generated additive Gaussian noise with N(0,2). As many noise vectors as the number of model parameters were extracted and added to them.
- Data-poisoning attack: A data-poisoning attack is a type of interference in learning using poisoned data and is known to be less effective than a model-poisoning attack. To evaluate the vulnerability of our system against a data-poisoning attack, the MNIST training data were manipulated by randomly generated additive noise with a Gaussian distribution N(10,5). We generated a random 28 × 28 noise image and added it to each MNIST training image.
- Label-swapping attack: A label-swapping attack, similar to a data-poisoning attack, is an efficient attack without directly damaging the model or training data. It proceeds simply by swapping the input labels for the corresponding data. For example, an attacker trains a model using 2 and 3 data, which are tagged by the swapped labels `3’ and `2’, respectively.

### 5.2. Shard Layer Review

We conducted two experiments to review the shard layer. First, we assessed the efficacy of similarity according to varying α. Next, we investigated the effectiveness of multiplicity.

#### 5.2.1. Performance According to Similarity Parameter α

**Figure 4:**
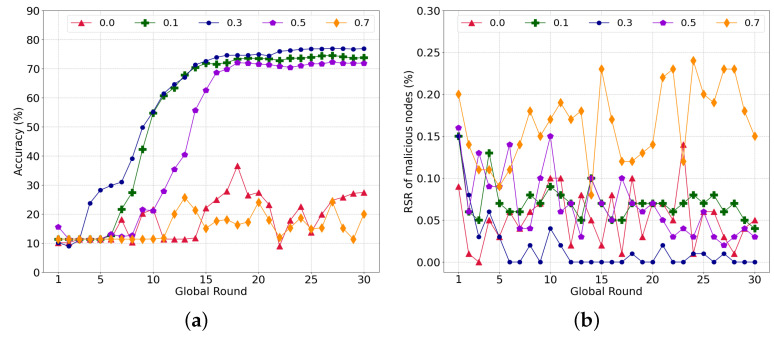
Performance subject to the various parameters α of similarity. (**a**) Global model accuracy; (**b**) Ratio of reference score of malicious nodes to total reference score per round.

To investigate the influence of similarity on the learning, we experimented with the local model aggregation by varying α in Equation (7). Here, 50% of the malicious nodes conducted a model-poisoning attack during federated learning. Figure 4a shows the accuracy of the global model with different α weights for the cosine similarity. First, federated learning failed to converge with α=0, which implies that it is difficult to distinguish between a poisoned model and a normal model without similarity. Because each worker only has data for two labels, the validation of the model is limited. Accordingly, malicious nodes can fine-tune the model weight to reduce the accuracy of a specific label or overfit the model using the data of a specific label. Meanwhile, the learning converged stably with α = 0.1, 0.3, and 0.5. Additionally, α = 0.3 achieved the highest accuracy of 76.84%, whereas accuracies of 73.75% and 71.85% were achieved with α = 0.1 and 0.5, respectively.

Figure 4b shows the ratio of the RS of malicious nodes to the total RS of all nodes. As the ratio is high, the nodes have more probability to select poisoned models. The RS ratio for α = 0.0 is comparable with the other cases, i.e., α = 0.1 and 0.5. In the case of α = 0.5 and α = 0.1, the average ratio of the RS of malicious nodes is 0.052% and 0.068%, respectively. However, the RS ratio with α = 0.3 is notably low compared to that of the others, which can lead the nodes to select normal transactions with a higher probability. Furthermore, the RS of the malicious nodes decreases as the training is repeated. As the nodes train the same global model using the given datasets repeatedly, the model change is limited, and the similarity increases along with the progress of the learning. Accordingly, the nodes can easily filter out the poisoned model through the similarity, which rapidly decreases the RS of the malicious nodes.

As illustrated in Figure 4a, less consideration of the accuracy with α = 0.7 increases the RS ratio and harms the global model with poisoned transactions, similar to α = 0.0. If a normal node selects a poisoned model, the weight of the normal node model becomes similar to that of the poisoned model. With a higher weight for the cosine similarity than the accuracy, nodes intend to continuously select a poisoned model if they previously selected a transaction of a malicious node owing to an immature global model or overwhelming malicious nodes.

From the above results, we assume that a sweet spot exists between the accuracy and similarity, such as at α = 0.3, which will be used for subsequent experiments.

#### 5.2.2. Multiplicity Effect on Performance

**Figure 5:**
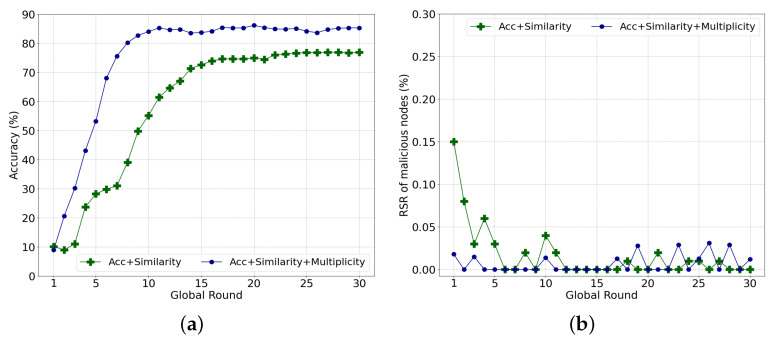
Effect of multiplicity on performance. (**a**) Global model accuracy; (**b**) Ratio of reference score of malicious nodes to total reference score per round.

To take advantage of the diversity of the data and models and avoid falling into a trap of similarity with malicious models, we propose using the multiplicity concept based on the structural characteristics of the DAG blockchain. Figure 5 demonstrates the effect of multiplicity in the same environment as the previous experiment. The tip selection algorithm using the accuracy and cosine similarity (i.e., the green line) converges to an accuracy of 76.84%. Meanwhile, the algorithm with an additional multiplicity converges to an accuracy of 85.24%. Additionally, the RS of a malicious node is lower when multiplicity is applied, as illustrated in Figure 5b. The multiplicity probably leads to the selection of malicious transactions for diversity even in higher rounds, which show some fluctuations in Figure 5b. However, it rarely affects a mature global model. Therefore, in the following experiments, we consider three aspects for the tip selection algorithm: the accuracy, cosine similarity, and multiplicity.

### 5.3. Main Blockchain Layer Review

Three experiments were conducted to investigate the main blockchain layer. First, we investigated its performance according to the proportion of the local model to the global model. Second, we compared different methods of model aggregation and voting. Third, the learning stability according to the average parameter β was compared.

#### 5.3.1. Performance Depending on the Local Model Proportion to the Global Model

We investigated the global model accuracy based on the combination of local models during the asynchronous update procedure.

In this experiment, only five nodes were used without malicious nodes to minimize incidental effects on the test results. Data sets were assigned to the nodes in the same manner as before.

**Figure 6:**
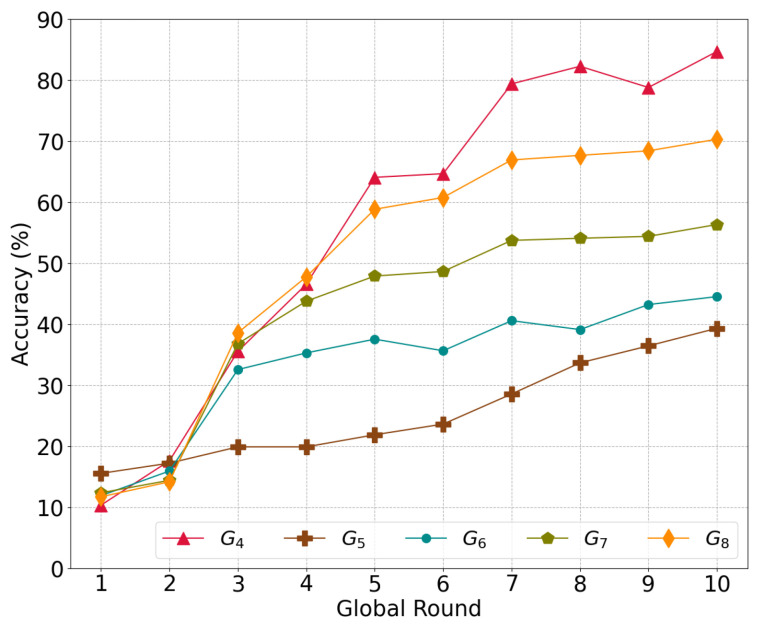
The accuracy of the global model depends on the proportion of local models to global models.

Figure 6 illustrates the convergence in accuracy according to the different numbers of local models aggregated in the global model $G_{m}$, where *m* indicates the number of local models used for aggregation. Essentially, the model $G_{|S|-1}=G_{4}$ with |S|=5 shards is a regular model that evenly aggregates all local models from each shard. Meanwhile, $G_{m>4}$ is an average global model that has an additional m−4 local models randomly chosen from among the five local models.

The base global model $G_{4}$ achieves a higher accuracy of 84.64% compared to the others; additionally, $G_{5}$ has the lowest accuracy of 39.29%, followed by $G_{6}$, $G_{7}$, and $G_{8}$. More information from the additional models leads to bias for particular classes in the global model and eventually decreases the level of accuracy, i.e., more iterations in a certain shard do not improve the accuracy of the global model

**Figure 7:**
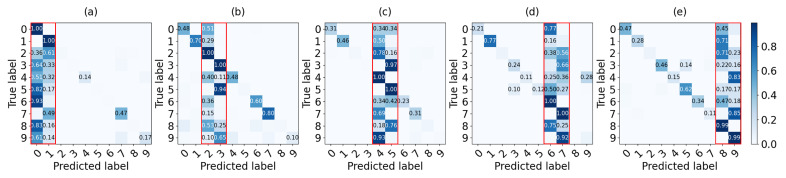
Confusion matrix of the $G_{4}$ global model added by a local model of each shard: (**a**) shard #1 with data 0–1, (**b**) shard #2 with data 2–3, (**c**) shard #3 with data 4–5, (**d**) shard #4 with data 6–7, (**e**) shard #5 with data 8–9.

Figure 7 presents the confusion matrix of global model $G_{5}$ with an additive model from shards 1 to 5. For instance, Figure 7a illustrates a case in which the local model of shard #1 for data 0 and 1 is added to $G_{4}$. Also, Figure 7b–e show confusion matrices of a global model combining a local model of each shard from #2 to #5 respectively. True positives and false positives for labels 0 and 1 are high as shown in the red box of each confusion matrix because the accuracy of other labels decreases as it contains more information about labels 0 and 1. Consequently, the non-IID data set leads the agents to train a model for classification more about their own data.

#### 5.3.2. Compare the Various Voting Systems

**Figure 8:**
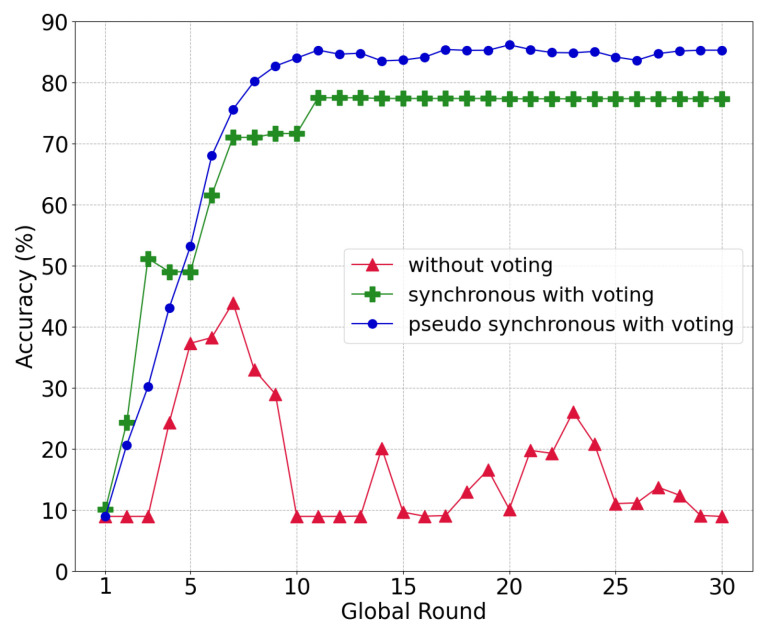
Global model accuracy under different voting methods.

Through this experiment, we demonstrated the effectiveness of the proposed voting system under a model-poisoning attack with 50% malicious nodes. Figure 8 displays the accuracy according to the voting method. Without voting, intentionally uploaded models of the malicious nodes as an attack can be aggregated in the global model, which imposes a serious effect on the entire learning procedure. Such a poisoned global model not only reduces the level of accuracy, it also degrades the ability to filter out other poisoned models in the local chain as the model similarity becomes meaningless. Therefore, the voting process is essential in circumstances in which a model attack from malicious nodes occurs in the network.

There are two aggregation-voting procedures: (i) a synchronous update for voting and (ii) a pseudo-synchronous update for voting. The first demands a synchronous aggregation of all local models from the shards. Thereafter, committee members vote between the old $G^{t-1}$ model and the new global model $G^{t}$. For example, if there are five shards in the network, the global model of the previous round is $G^{t-1}=\frac{M_{1}^{t-1}+M_{2}^{t-1}+M_{3}^{t-1}+M_{4}^{t-1}+M_{5}^{t-1}}{5}$, and the global model of the current round is $G^{t}=\frac{M_{1}^{t}+M_{2}^{t}+M_{3}^{t}+M_{4}^{t}+M_{5}^{t}}{5}$. The synchronous approach has no significant change in accuracy from round 11, which converges at approximately 77.3% because the new global model is comparable with the old global model.

The pseudo-synchronous update for voting (described in Section 4.4) achieves an accuracy of up to 85.85% and a convergence of approximately 85.24%. In contrast to the synchronous case, which discards a new global model even with a single poisoned local model, the pseudo-synchronous approach can filter out the poisoned local model while combining other pure local models for the new global model.

#### 5.3.3. Global Model Update Speed β

**Figure 9:**
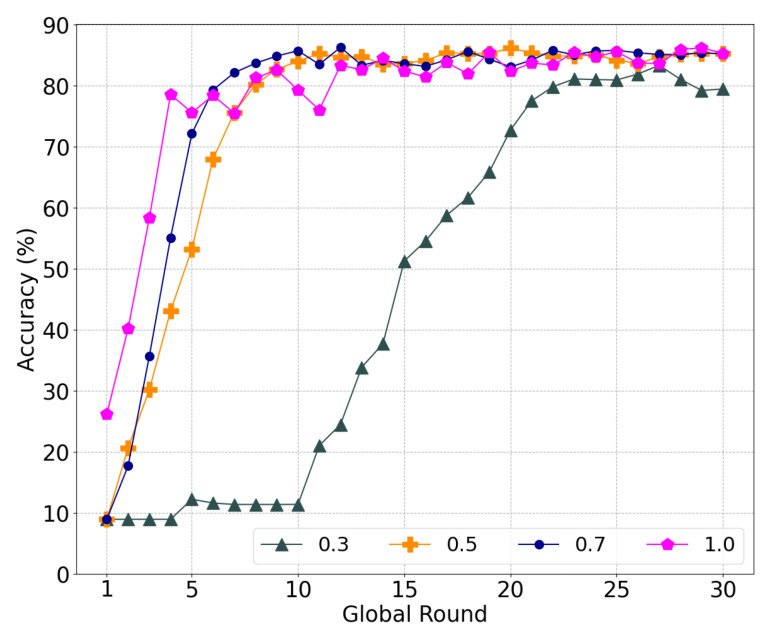
The accuracy of a global model depends on the adjustment of β.

Our global model was progressively averaged using $\omega^{\left(t+1\right)}=\left(1-\beta \right)\omega^{\left(t-1\right)}+\beta \omega^{\left(t\right)}$ for a gradual update, where β is a ratio parameter between the old and new global models. Figure 9 plots the accuracy by varying β under 50% malicious nodes for a model-poisoning attack. The accuracy of β = 1.0 reaches a maximum of 85.26%, whereas other β values of 0.5, 0.7, and 0.3 achieve accuracy rates of 85.24%, 85.15%, and 79.41%, respectively. However, β = 1.0 considering only a new global model shows unstable learning. In particular, the accuracy decreases, particularly in global rounds 5, 7, 10, 11, 16, 18, and 20, because some of the local models used for global model aggregation were indirectly poisoned. Particularly in the initial rounds, normal nodes have difficulty distinguishing between poisoned and normal models because of the model variance, which exacerbates the stability in terms of accuracy.

With a small β = 0.3, the previous global model was reflected more than the new global model, which made the learning extremely slow. We therefore used an update speed of α = 0.5 for the following experiments because stable learning with an accuracy comparable to β = 1.0—the accuracy gap between them is only 0.02—is achieved.

### 5.4. Performance for the Various Attack Scenarios

The following experiments demonstrate the feasibility of our system under various attacks. Unlike the previous model poisoning, these two data-poisoning and label-swapping attacks affect the model indirectly.

For comparison, the conventional FedAvg was implemented with 20 epochs of training per round, which is equivalent to our training iterations. Additionally, we benchmark several Byzantine- tolerant algorithms such as Median, TrimmedMean [12], and Krum [11] in model-poisoning attack scenarios.

#### 5.4.1. Model-Poisoning Attacks

**Figure 10:**
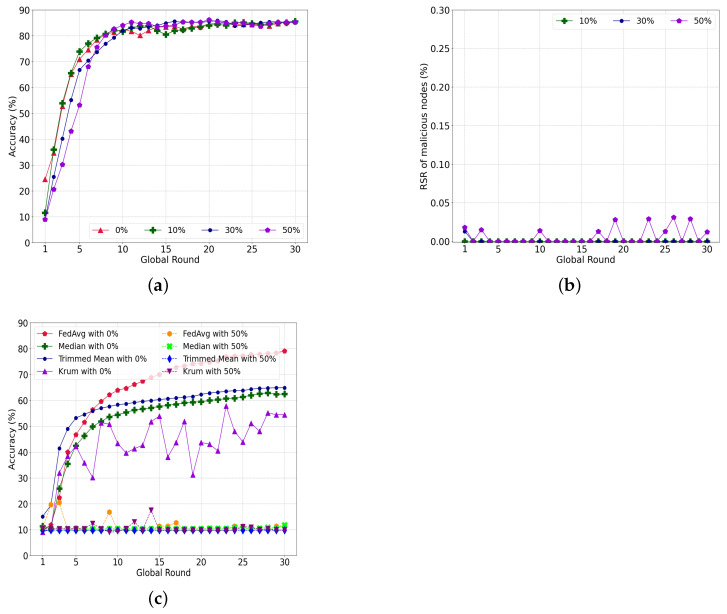
Evaluation of our system with a model-poisoning attack. (**a**) Performance of the proposed system; (**b**) RSR with varying ratio of attackers; (**c**) Performance comparison of FedAvg, MedianTrimmedMean and Knum.

Figure 10a shows the accuracy of the performance with varying ratios of malicious nodes attempting a model-poisoning attack. The final accuracy of each case, i.e., 0%, 10%, 30%, and 50% malicious nodes, is 85.67%, 85.5%, 85.07%, and 85.24%, respectively. The accuracy of the convergence is comparable regardless of the ratio of malicious nodes. Instead, the learning speed differs according to the proportion of malicious nodes. A small portion of malicious nodes, i.e., 0% and 10%, learned faster than the 30% and 50% cases. Moreover, the accuracy is 24.47% in round 1 with no malicious nodes, i.e., 0%, compared to other cases such as 10%, 30%, and 50% malicious nodes. There are two key reasons for the slow learning with a large number of malicious nodes. First, the number of honest nodes decreases in each shard. The second is that the partially poisoned global model, which is given as a root transaction at every round, delays updates in each shard. To summarize, the malicious nodes do not significantly affect the accuracy of our system but cause latency in the model updated for the target.

Figure 10b depicts that a malicious node does not effectively attack the learning of a normal node. The RS of the malicious nodes is almost consistent with the varying ratios of malicious nodes, less than 0.05% for all cases. The case with 10% malicious nodes has an RS sum of almost 0%, while the case with 30% malicious nodes has an RS sum of 0.013% in round 1. For a 50% ratio of malicious nodes, the influence of malicious nodes is insignificant, with 0.05% RS.

Figure 10c shows the comparison of Byzantine-tolerant algorithms together with the conventional FedAvg with respect to accuracy performance. All experimental parameters are configured to be exactly the same as those in Figure 10a. Without malicious nodes, the FedAvg accuracy is about 79%, while the highest average accuracy is only about 10%. Moreover the training fails to converge to the global model with 50% malicious nodes. The final accuracy of Median, TrimmedMean, and Krum is 62.4%, 65.83%, and 55.4%, respectively, without attacks. Unlike the FedAvg using the model weights of all client nodes, those Byzantine-tolerant algorithms use only a fraction of the model weights of client nodes, which makes learning more unstable on non-IID datasets. Thus, all three algorithms have lower accuracy than FedAvg. Additionally, all three algorithms are not trained with 50% malicious nodes because simple clustering algorithms or statistical approaches cannot effectively distinguish the local models that have been biased by the non-IID datasets and poisoned by attackers.

#### 5.4.2. Data-Poisoning Attacks

**Figure 11:**
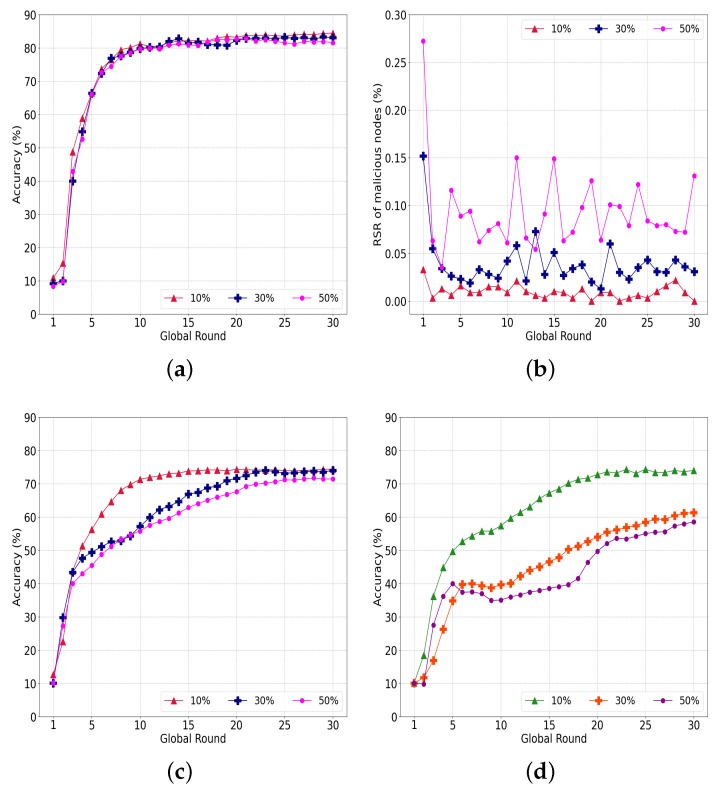
Evaluation of our system under data-poisoning attacks. (**a**) Performance of the proposed system; (**b**) RSR with varying ratio of attackers; (**c**) Performance of legacy FedAvg; (**d**) Performance of less-trained FedAvg model.

Figure 11a shows the accuracy of our system with various malicious nodes attempting data-poisoning attacks. In contrast to the model-poisoning attack having 85% accuracy regardless of the portions of malicious nodes, the accuracy converges to 81.53%, 83.03%, and 84.41% when the ratio of malicious nodes is 50%, 30% and 10%, respectively. Our system has limitations in detecting a model ruined by poisoned data compared to directly filtering a poisoned model.

The RSR of the malicious nodes in Figure 11b indicate similar outcomes. The RSR is 0.01%, 0.04%, and 0.09% for 10%, 30%, and 50% malicious nodes, respectively. In Figure 11b, the RSR decreases sharply in rounds 1 and 2, and the interference of malicious nodes is sufficiently reduced to allow the learning to converge as the learning progresses.

Figure 11c shows the accuracy of a conventional FedAvg under the same data poisoning. Unlike the model-poisoning attack, where the accuracy could not converge, the data-poisoning attack converged to 74.37% when the number of malicious nodes was 10%. The accuracy converged to 73.99% and 71.42% with 30% and 50% malicious nodes, respectively. Consequently, our system outperformed the conventional FedAvg by an average of 9.72%.

To explore more interference from data poisoning, we conducted an additional experiment with a reduction in the number of epochs of each round from 20 to 5, as indicated in Figure 11d. With 10% malicious nodes, the accuracy converges to 73.95%, which is similar to that of 20 epochs and implies that a small number of malicious nodes do not significantly affect the federated learning. However, the accuracy degrades to 61.28% and 58.49% for more than 30% and 50% malicious nodes, respectively. From these results, we can assume that the accuracy can be improved if the nodes have a sufficient number of training epochs. In the case of a data-poisoning attack in which the weights of the model are not directly manipulated, a sufficient number of training epochs can enhance the resistance to an attack.

#### 5.4.3. Label-Swapping Attacks

**Figure 12:**
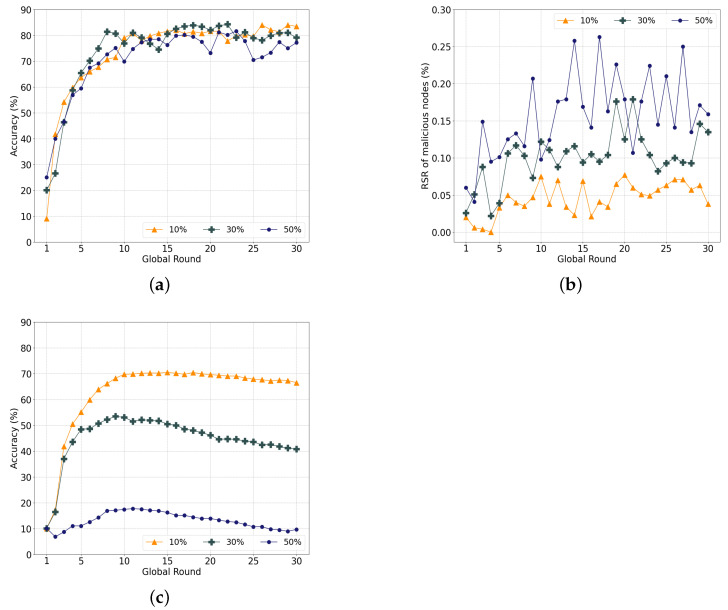
Evaluation of our system with label-swapping attacks. (**a**) Performance of the proposed system; (**b**) RSR with varying ratios of attackers; (**c**) Performance of legacy FedAvg.

The label-swapping attack influences learning more than the data-poisoning attack by indirectly damaging the weights of the model. Figure 12a illustrates the accuracy with varying ratios of malicious nodes that conduct label-swapping attacks. When there are 10% malicious nodes, the accuracy in the last round is approximately 83.41% and decreases sharply to 79.08% with 30% malicious nodes. Under 50% malicious nodes, the accuracy is approximately 77.16% in the last round.

Unlike previous experiments, there is a gap in the level of accuracy in the last round according to the ratio of malicious nodes. Additionally, the RSR of malicious nodes increases and continuously fluctuates significantly, as indicated in Figure 12b, which implies that the attacks continuously affect the entire learning procedure. Unlike data-poisoning attacks, label-swapping attacks use training data without noise, similar to normal nodes, which makes it difficult to distinguish poisoned models using the cosine similarity. Therefore, the models of malicious nodes are continuously selected, as indicated in the figure.

Nevertheless, our system achieves a notable accuracy with a two-step verification that selects the current and previous local models through voting. Although the malicious node model is directly reflected in the local model, it is possible to prevent the global model from being severely damaged.

Figure 12c displays the performance of the conventional FedAvg, where the accuracy converges to 66.5% for 10% attackers and 40.75% and 9.8% for 30% and 50% malicious nodes, respectively. The accuracy decreases significantly according to the ratio of malicious nodes. The adverse effect on the learning is lower than that of the model-poisoning attack but higher than that of the data-poisoning attack.

## 6. Conclusions

In this article, we present a hierarchical blockchain system for robust federated learning under various attacks by the malicious nodes, which consists of a main blockchain layer for pseudo-asynchronous global model aggregation and a DAG-based shard layer for local model aggregation. We propose a novel tip selection algorithm with local model similarity and a multiplicity concept that prevents effectively falling into traps of similarity attacks by malicious nodes. Additionally, the proposed global voting algorithm achieves a consistent improvement in the model accuracy under various attacks and an extremely non-IID data distribution. We implement our framework and experiment with several attack models. Our system outperforms non-IID FedAvg learning with better accuracy, 60, 10 and 60% in the model-poisoning, data-poisoning and label-swapping attacks, respectively. Additionally, some Byzantine-tolerant algorithms proposed for the model-poisoning attack show limited throughput as they suffer when distinguishing and aggregating local models due to the non-IID distribution of the dataset.

## 7. Future Works

In this study, we find optimal parameters for similarity, accuracy and update speed by exploring experiment sets for given blockchain structures and datasets. However, they need to be configured automatically during the learning procedure; therefore, in the future, we will apply a deep reinforcement learning algorithm for parameter tuning. Additionally, we will investigate the feasibility of our framework for more complicated CNN models for rich image data processing and recurrent network models for time series data. In our system, the model aggregation causes heavy communications and storage overuse for the blockchain. Accordingly, further studies on scalability enhancement need to be conducted.

## Figures and Tables

**Table 1:** The list of symbols that are used to describe the proposed system.

Notation	Description
N	Set of client nodes in a federated learning network.
*i*	A client node that conducts local training in the network. i∈N
$D_{i}$	Set of local data samples that each node *i* owns.
η	Learning rate.
*w*	The weight of model.
$\zeta_{j,k}$	The cumulative reference score (CRS) of the transaction.
*S*	Set of shards in the network.
$S_{k}$	A shard where the index is *k*.
$M_{t}^{s}$	The local model for the maximum reference score within a specific range in each shard.
$G_{\rho }^{t}$	A global model.
ρ	The integer value starting from 0 to |S| as the order of the shard models uploaded to the main blockchain.
V	Voting function: nodes belonging to the committee select one model based on the accuracy of the two models.
*r*	Global round
*B*	The local batch size
*E*	The number of local epochs
TX	A transaction in the DAG blockchain.
